# Single and Combined Methods to Specifically or Bulk-Purify RNA–Protein Complexes

**DOI:** 10.3390/biom10081160

**Published:** 2020-08-07

**Authors:** Roosje Van Ende, Sam Balzarini, Koen Geuten

**Affiliations:** Molecular Biotechnology of Plants and Micro-organisms, KU Leuven, Kasteelpark Arenberg 31, 3001 Leuven, Belgium; roosje.vanende@kuleuven.be (R.V.E.); sam.balzarini@kuleuven.be (S.B.)

**Keywords:** ribonucleonprotein complexes, RNA-binding proteins, phase separation, RNA-centric

## Abstract

The ribonome interconnects the proteome and the transcriptome. Specific biology is situated at this interface, which can be studied in bulk using omics approaches or specifically by targeting an individual protein or RNA species. In this review, we focus on both RNA- and ribonucleoprotein-(RNP) centric methods. These methods can be used to study the dynamics of the ribonome in response to a stimulus or to identify the proteins that interact with a specific RNA species. The purpose of this review is to provide and discuss an overview of strategies to cross-link RNA to proteins and the currently available RNA- and RNP-centric approaches to study RNPs. We elaborate on some major challenges common to most methods, involving RNP yield, purity and experimental cost. We identify the origin of these difficulties and propose to combine existing approaches to overcome these challenges. The solutions provided build on the recently developed organic phase separation protocols, such as Cross-Linked RNA eXtraction (XRNAX), orthogonal organic phase separation (OOPS) and Phenol-Toluol extraction (PTex).

## 1. Opening Doors to the RNA-Binding Proteome

### 1.1. Why Study These Proteins? What New Exciting Insights Can Such Study Reveal?

Ribonucleoprotein complexes (RNPs), composed of both RNA molecule(s) and one or more RNA-binding proteins (RBPs), are known to play key roles in cell homeostasis and cell fate by controlling post-transcriptional gene regulation and RNA processing. This can involve gene expression, RNA storage, RNA stability and RNA transport [1]. Until recently, RBPs were classified as such by the presence of one or more RNA-binding domains (RBDs), such as an RNA-recognition motif (RRM), K homology domain (KH), DEAD-box helicase domain, Pumilio/FBF domain, zinc fingers, etc. [2]. However, several research groups have discovered hundreds of RBPs missing such domains, suggesting the limitations of computational prediction [3,4]. The discovery of RBPs lacking any canonical RBD underscores that the number of RBPs is likely underestimated [5,6]. It has been shown that cells adapt to physiological cues through changes in the RBPome, making the study of the dynamics of the RBPome a starting point for many biological questions [7,8,9]. The study of these RNPs could provide important insights into, for example, the regulation and function of long noncoding RNAs (lncRNAs). The mechanism of how Xist, one of the most extensively studied lncRNAs, regulates the silencing of the X-chromosome was recently elucidated by the study of its interacting RBPs [10,11]. Furthermore, the abnormal functioning or expression of RBPs can be linked to multiple diseases [12], as was demonstrated for the hereditary hyperferritinemia-cataract syndrome, arising from a mutated RBP [13]. Additionally, the identification of viral susceptibility genes by studying RNPs could provide new perspectives, as was shown by several research groups [9,14,15,16,17,18]. Opening doors to the RNA-binding proteome is resulting in exciting new insights into a rapidly evolving scientific field.

### 1.2. The Study of the Ribonome: Where Are We Now?

Over the last two decades, several techniques have been developed to study the interplay between RNA and their cognate RBPs. These methods use an RNA-centric or protein-centric approach and target a specific RNP or an entire group of RNPs. For such a rapidly evolving field, it can be challenging to maintain an overview of the available methods and more importantly how they can be applied. Additionally, standard ways of analyzing, presenting and visualizing the resulting data have yet to emerge.

It has become clear that RNA is more than solely a blueprint to be translated into functional proteins, as it can also function as a scaffold to coordinate and organize protein networks and vice versa. Earlier, protein-centric approaches, such as Cross-Linking and ImmunoPrecipitation (CLIP), were applied to describe these interactions [19,20]. Although successful, these approaches are focused on a single species of protein interacting with one or a diverse group of RNA molecules. However, the question is often complementary but reversed: which proteome interacts with an RNA species?

Despite the recent availability of multiple RNA-centric methodologies discussed in Section 3, only a few specific RNA molecules have been studied extensively, of which the resulting data did not always yield consistent results. Not only does each method have its own strengths and weaknesses, but there are some challenges, involving yield, cost and specificity, common to all. Overcoming these could result in major advances in the field and a better characterization of multiple RNPs, even the ones difficult to study, such as those associated with low-copy-number RNAs. With the recent publication of organic phase separation protocols such as Cross-Linked RNA eXtraction (XRNAX) [21], orthogonal organic phase separation (OOPS) [22] and Phenol-Toluol extraction (PTex) [23], we address these challenges and propose future experimental strategies that build on these methods.

As mentioned in Urdaneta et al. (2019) and Beckmann (2017), we have to highlight that the term RBP has a historical role in functional RNA biology. Since cross-linking an RNP is based on the physical proximity of the RNA and the protein rather than on the physiological role of the protein, the term RBP becomes broader or a new term has to be introduced, as suggested by Urdaneta et al. Because of this proximity binding, the question of whether the identified binding proteins are functional binders or just spatially close to a given RNA molecule during the time of cross-linking can arise. Together with the fact that a lot is still unknown about the role and importance of numerous transient RNA–protein interactions, it is advisable to experimentally validate identified targets, no matter how stringent the purification procedure [23,24].

## 2. Cross-Linking Proteins to RNA or Not, and How?

**Figure 1:**
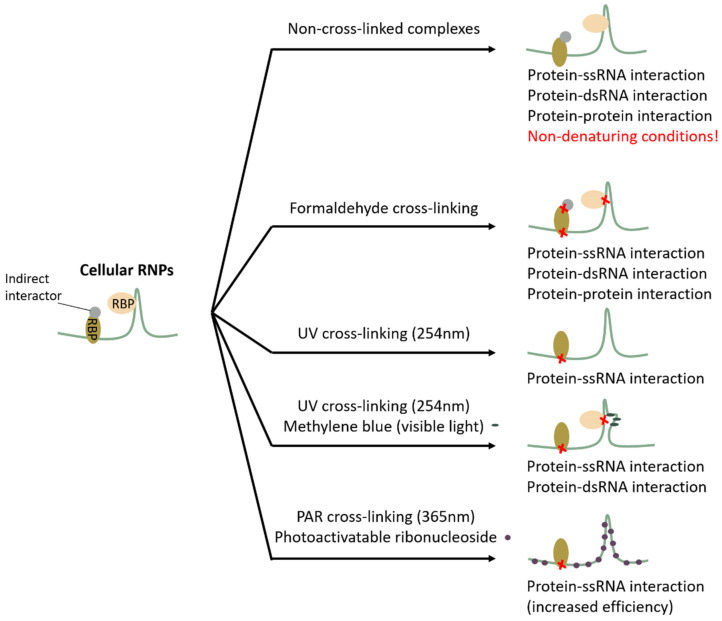
Overview of cross-linking strategies. Depending on the sample type and goal of the set-up, no cross-linking, chemical- and UV-induced cross-linking could be used. Cross-links are marked with a red cross. Formaldehyde cross-linking induces RNA–protein cross-links (both double-stranded as single-stranded), as well as protein-protein cross-links. In contrast, UV light (254 nm) only induces protein–single-stranded RNA molecule (ssRNA) cross-links. Substituted methylene blue will open up a double-stranded RNA (dsRNA) structure by intercalation, allowing dsRNA protein binders to be cross-linked using visible light. The combination of methylene blue (visible light) and UV light (254 nm) induces both ssRNA–protein and dsRNA–protein cross-links. PAR (photoactivatable ribonucleoside-enhanced) cross-linking (365 nm) is a variant of conventional UV cross-linking, increasing cross-linking efficiency due to the incorporation of a photoactivatable nucleoside analog. Figure adapted from Wheeler et al., (2018) [25].

A first step when embarking on a study of the ribonome is to choose whether or not a covalent bond will be introduced between the RNA molecule and its interacting proteins. Depending on the sample type and goal of the set-up, no cross-linking, chemical- and UV-induced cross-linking could be used. An overview of the strategies is provided in Figure 1.

### 2.1. Non-Cross-Linked Complexes: A Good Idea?

Isolating RNPs in vivo in their native state under physiological conditions is as close as it gets to the true nature of these complexes and therefore, if successful, harbors the most accurate and desirable information. Nevertheless, these native conditions have several practical challenges. The native RNP is as strong as its weakest non-covalent interaction, and therefore mild lysis conditions and washing procedures have to be used to maintain these interactions. These washing conditions do not discriminate between protein–RNA and protein–protein interactions, leaving secondary binding proteins in the RNP complex and leaving non-RNA binding protein contaminants to be co-purified. Additionally, aspecific RNPs can re-associate after cell lysis, making the discrimination between specific and contaminating interactions more difficult [26]. Under native conditions, the RNA molecule will harbor secondary structures, complicating RNA-centric antisense purification approaches. When proven successful, the protein–RNA interactions identified with these methods often require further experimental validation. However, to obtain an enhanced signal-to-noise ratio, both chemical and UV cross-linking techniques have been developed, enabling more stringent isolation.

### 2.2. Formaldehyde as a Promiscuous Cross-Linker

Chemical cross-linking is a widespread technique used successfully for multiple protein- and RNA-centric approaches [27]. Often the chemical of choice is formaldehyde, which is a small bifunctional and reversible cross-linker that can easily permeate cell walls and membranes to reach all the necessary cell compartments. To provide a good balance between the protein–RNA cross-linking and solubility of the complexes, the optimal concentration and fixation time of formaldehyde are cell- and tissue type-dependent and should be optimized as described in Patton et al., (2020) [28]. Although less often used, diepoxybutane [29,30] and nitrogen mustard [31] have also been used as RNA–protein chemical cross-linkers to cross-link RNPs. As an advantage, chemical cross-linking can be used to identify the binding sites of proteins that photo-cross-link poorly with RNA. On the other hand, from an RNA-centric view, chemical cross-linking compounds also efficiently introduce protein–protein and protein–DNA cross-links to a much higher extent compared to UV cross-linking [32]. This makes it more difficult to distinguish the RNA–protein interactions from the rest of the formed macromolecules, potentially introducing false-positives. Chemical cross-linking could be used for tissues where UV light cannot penetrate the whole tissue. However, the recently noticed property of frozen powdered tissue, which can be used as source material for UV cross-linking, could circumvent this problem [23]. In summary, chemical cross-linking and UV cross-linking are complementary techniques and can be useful RNA-centric methods to use as a first identification step, followed up by protein-centric approaches to verify whether the protein has the expected RNA binding property.

### 2.3. UV Light as a Specific but Low-Efficiency Cross-Linker

Ultraviolet light (UV) promotes the formation of an irreversible covalent bond between a single-stranded RNA molecule (ssRNA) and its interacting RBPs [33]. To produce this bond, the amino acid and nucleotide have to be within the Ångstrom distance, limiting the introduction of off-target bond formation, which could introduce false-positives [34]. Importantly, this covalent bond makes the purification of RNPs possible in a harsh denaturing environment, limiting potential contaminants. Both DNA and double-stranded RNA (dsRNA), however, have less accessible nucleotide bases and are therefore less likely to cross-link. A potential approach to overcome this issue is the use of methylene blue. This molecule intercalates between the interacting bases, resulting in them becoming more accessible [35]. Complementary to methylene blue, it is shown that the addition of additives such as 2-iminothiolane [36,37] and dithiothreitol (DTT) [38] can also form efficient and selective UV-inducible cross-links. This is worthwhile to further investigate.

Conventional UV cross-linking uses a wavelength of 254 nm, and both laser light and UV lamps can be utilized to introduce enough energy to form the covalent link [39]. Specific biophysical and chemical events upon UV-mediated cross-linking are well explained in Urdaneta et al. (2019) [23]. It is estimated that only 1–5% of the interacting proteins are successfully cross-linked, illustrating a major drawback of this cross-linking technique [40]. To increase this percentage, PAR (photoactivatable ribonucleoside-enhanced) cross-linking has been developed. PAR cross-linking makes use of introducing photo-activatable nucleoside analogs into the RNA which can be photo-activated using 365 nm light. Although many existing photoactivatable nucleoside options are available (reviewed in [33]), 4-thiouridine (4SU) and 6-thioguanosine (6SG) analogs are the most commonly used. By using longer wavelengths (365 nm), less UV damage is thought to be induced, which can be an important issue, depending on the goal of the experiment. PAR cross-linking has shown to yield 100–1000 times more cross-linked complexes compared to 254 nm UV light [41]. Unfortunately, these nucleoside analogs are only usable if they are not toxic to the cells, are efficiently metabolized into its RNA and do not influence the cell’s normal state, limiting their approach to a subset of cell cultures and excluding most tissues.

Amino acids differ in structure and therefore also in preference to form a covalent link with their interacting nucleotides. A study by Kramer et al., 2014 found that all amino acids, with the exception of Asp, Asn, Glu and Gln, could cross-link with RNA. Cys, Lys, Phe, Trp and Tyr are cross-linked with a greater frequency than other amino acids [42]. Asp and Glu are negatively charged, and therefore it is not surprising that these two amino acids do not interact with negatively charged RNA. Why Asn and Gln are not found to form a link is yet to be determined. Another study focusing on the binding site found similar amino acids to have a preference to cross-link [43]. Interestingly, a Gene Ontology (GO) study comparing two RBPomes obtained by 254 and 360 nm (PAR) highlighted a clear difference in the recovery of proteins that lack known functions in RNA metabolism. These proteins were recovered more often when using 254 nm UV light. Shchepachev et al., 2019, hypothesized that this difference lies in the properties of the nucleotides activated by the UV light [43]. Native nucleotides upon irradiation absorb photons and will very rapidly release this energy as heat to prevent damage, enabling the repetition of this process [44]. In contrast, 4-thiouridine forms a reactive triplet upon the absorption of the photons [45]. If the triplet is not cross-linked to a suitably positioned amino acid, uridine or uridine sulfonate will be formed, and this will terminate the potential of this 4-thiouridine to be cross-linked. Since native nucleotides irradiated by 254 nm UV light have multiple opportunities for cross-linking, transiently interacting proteins will have an increased chance to be cross-linked in comparison with 4-thiouridine using 360 nm UV light [43]. This hypothesis is consistent with other studies identifying RBPs using both 254 nm and 365 nm which found both overlapping and wavelength-specific proteins, indicating that both techniques are rather complementary and could cross-link different binding sites [24].

Before UV light can perform a cross-link, it has to reach the RNPs, which makes this approach variable between tissues. Translucent tissue types or single cell cultures or organisms will absorb UV light more easily and therefore are more amenable to UV cross-linking. It is not surprising that the first generation of RNP identifications making use of UV light has been performed on single cell cultures (*Homo sapiens* cell lines [3,7], *Mus musculus* [46,47], *Arabidopsis thaliana* protoplasts and cell cultures [39,48,49]), single cellular organisms (*Trypanosoma brucei* [50], *Leishmania* [51], *Plasmodium falciparum* [52], *Saccharomyces cerevisiae* [24,53,54,55]) and organisms containing a low number of cells or embryogenic tissue (*Caenorhabditis elegans* [53], *Mus musculus* [46], *Drosophila melanogaster* [56], *Danio rerio* [57]). The different studies make use of UV doses between 0.2–3 J/cm^2^. The perfect dose of UV light is likely cell/tissue-dependent and should be optimized before applying. It is seen that an overexposure to UV light can decrease the yield of RNPs, likely due to UV-triggered protease digestion or RNA degradation [23]). A balance between sufficient cross-linking and avoiding UV-overexposure has to be found to keep the cellular state as close as possible to physiological conditions.

Applying UV cross-linking to plant tissue appears to be more challenging due to the presence of a cell wall, the thickness of the leaves and the presence of UV-absorbing pigments and additional secondary metabolites [58]. However, recently less light-permeable tissues or high cell number tissue (e.g., etiolated seedlings [59], *Arabidopsis thaliana* leaf tissue [58,60], mouse brain [61]) have been successfully used for cross-linking and have identified RBPs, confirming UV light as a tool for cross-linking multilayer tissues. Notably, Urdaneta et al. (2019) showed that frozen mouse brain tissue ground into powder could be UV cross-linked in a frozen state to investigate the RBPome. This method opens the door to theoretically every type of tissue to be UV-cross-linked, making the study of all types of in vivo tissue possible.

## 3. Overview of Available RNA- and RNP-Centric Purification Methods

In this review, we will mainly focus on RNA- and RNP-centric in vivo methods to identify RNA-binding proteins. In vitro, in silico and in vivo protein-centric methods, which have been equally successful, fall outside the scope of this review and are more elaborately reviewed in [62].

### 3.1. Isolating the RNA-Bound Proteome to Study RNP Dynamics

Multiple techniques are available to target the RBPome. These methods can be categorized into three main groups: affinity-based-, solid-phase-based and based on organic phase separation. A summary can be found in Table 1.

#### 3.1.1. Affinity-Based Separation

##### (Enhanced/Comparative) RNA Interactome Capture ((e/c) RIC)

RNA Interactome Capture (RIC) was the first RNA-centric approach developed to address the limitations of in vitro, bio-informatic and protein-centric approaches to identify the mRNA interactome without prior knowledge of the RNA interacting proteins. Cellular RNPs are UV cross-linked, after which the mRNA-bound proteome is purified using oligo(dT) beads and identified using quantitative mass spectrometry [3,4]. Once a protein is enriched in an irradiated sample compared to the non-cross-linked control sample, it is defined as an RBP. Data obtained using this methodology reveal that the eukaryotic mRNA-binding proteome is substantially larger than anticipated. Although RIC is an important technique to identify the mRNA interactome, it also has several drawbacks. Only proteins interacting with poly(A) tailed RNA are captured, and thereby they represent only a part of the whole RBPome, leaving out rRNA-, tRNA-, ncRNA-, lncRNA-, snoRNA- and snRNA-interacting proteins. Consequently, RBPomes of prokaryotes lacking abundant poly(A) tails cannot be isolated using RIC. In addition, the technical noise and experimental variability of RIC still limit the utility of the technique to study the function and dynamics of the RBPome upon environmental and pharmacological stimuli. To deal with these variation issues, enhanced RIC (eRIC) using Locked Nucleic Acid (LNA)-modified probes, allowing the use of more stringent washing conditions, was designed. This optimized protocol reduces the material requirement, improves signal-to-noise ratios (10 times less rRNA and DNA contamination was observed) and enables studying the dynamics of the mRNA interactome upon different experimental conditions [7]. In addition, Garcia-Moreno et al. (2019) developed comparative RIC (cRIC), by altering the existing RIC protocol with the use of SILAC (stable isotope labelling by amino acids in cell culture), enabling the study of the dynamics of the mRNA interactome upon sindbis viral infection. It was shown that a quarter of the mRNA interactome changes upon sindbis infection, of which a few were proven to play a vital role in viral virulence. This research clearly shows the usefulness of RNP capture methods in studying physiologically important systems and cues [9].

##### RNA Interactome Using Click Chemistry (RICK) and Click Chemistry-Assisted RNA-Interactome Capture (CARIC)

To complement the shortcomings of RIC to capture RNA molecules not harboring a poly(A) tail, multiple methods were designed. Of these, click chemistry-assisted RNA-interactome capture (CARIC) and RNA interactome using click chemistry (RICK) were among the first [68]. RICK and CARIC share a similar approach, only differing in the addition of the photoactivatable 4-thiouridine (4SU) nucleotide analogs and, consequently, also the wavelength of UV cross-linking by CARIC. Instead of targeting a specific RNA sequence or motif, a nucleoside analog (5-ethynyluridine (EU)) is built into the RNA and chemically linked with biotin after a UV cross-linking step [68]. The biotin-linked RNPs can now be isolated using streptavidin-coated magnetic or agarose beads. Since the nucleoside analog is incorporated in all newly transcribed RNA species, a more complete set of the total RBPome is captured in comparison with RIC, which only gathers the poly(A) tailed fraction of the RBPome. Since nucleoside analogs can only be built into newly transcribed RNA from the moment they are administered to the cell culture, one can selectively study the newly transcribed fraction of RNP interaction upon a certain stimulus over time. This feature distinguishes the click chemistry RNA interactome from all other strategies. However, since click chemistry requires the efficient in vivo labelling of RNA, its application may be limited to suitable cell cultures, thereby narrowing its scope. Besides, it should be noted that the streptavidin-based purification can result in the co-purification of rare naturally biotinylated proteins, affecting the reliability of the output.

#### 3.1.2. Solid-Phase Separation

##### Complex Capture or 2C Method

The power of the 2C method lies in its simplicity. The method is based on the well-established principle of silica matrices to strongly and specifically retain nucleic acids [69]. Asencio et al. (2018) observed that the silica–RNA interaction is sufficiently strong to also retain UV cross-linked RNA–protein complexes [66]. Since the silica matrix interacts with the RNA molecule in a sequence-independent manner, the whole RBPome is purified. Although no mass spectrometry identification was performed, the bioanalyzer and silver staining results of the retained, washed and eluted RNP fraction points to an enriched level of good integrity RNA–protein complexes. Despite the silver staining showing that large protein bands (range 250 kDa) are co-eluted, more investigation is necessary to elucidate whether all size and/or sorts of biochemically different RNPs are purified with the same efficiency. The XRNAX protocol (discussed later) circumvents this potential problem by pre-treating the complexes with partial trypsin digestion, degrading parts of the protein [21]. The resulting smaller RNPs are probably retained more efficiently and in a more quantitative way, which is essential for the study of RBPome dynamics. Asencio et al. (2018) envisage the use of 2C beyond the validation of RBPs but also to simplify multiple downstream applications to study RNA–protein interactions. By introducing multiple additional purification steps, the RNA-binding domain map (RBDmap), RBPome, RNA binding protein footprint (RBP footprint), RNA interactome and a targeted RBDmap of a single protein could be studied further, as discussed in Section 4.

##### Total RNA-Associated Protein Purification (TRAPP) and iTRAPP

Similar to the 2C method, TRAPP also makes use of a silica solid phase to retain the RBPome in a non-sequence-specific manner [43]. Instead of silica columns, silicon dioxide particles of 0.5–10 µm are used. The identification of the RBPome of *Escherichia coli* and *S. cerevisiae* was successful and, in addition, Shchepachev et al. could map the dynamics of the RBPome of *S. cerevisiae* upon weak acidic stress using this technique. Silica matrices are known to also retain DNA, possibly giving rise to DNA-interacting contaminants. The combination of the low DNA-protein linking capacity of UV light, denaturing conditions and an extra washing step with a low salt buffer containing 80% ethanol reduces the recovery of contaminating proteins bound to DNA, as clearly shown by the authors. For *S. cerevisiae* samples, different UV types and doses (400/800/1360 mJ/cm^2^ at 254 nm and 7200 mJ/cm^2^ at 360 nm) were compared for both RNP recovery and for the pathways in which identified proteins function (GO analysis). The quantitative mass spectrometry results clearly show a UV dose-dependent enrichment of RNPs (573/694/1434 proteins) using 245 nm with an overlapping core of 482 proteins in the three UV conditions (400/800/1360 mJ/cm^2^). The authors also investigated the RBPome composition using 254 nm (TRAPP) and 365 nm (PAR-TRAPP) UV cross-linking light. Interestingly, GO analysis shows a clear difference for proteins lacking known functions in RNA metabolism/binding, which were more abundant in TRAPP compared to PAR-TRAPP, highlighting the complementary linking character of both UV approaches, as discussed in Section 2.2. Additionally, they could also pinpoint the precise interaction site of 524 RNA-peptide cross-links belonging to 178 proteins using an adapted version of the Xi search engine software [70], coining this approach, iTRAPP.

##### Viral Cross-Linking and Solid Phase Purification (VIR-CLASP)

VIR-CLASP focusses on the identification of the pre-replicated interactome of theoretically all mammalian RNA viruses [67]. In short, the virus is propagated in host cells substituted with 4SU nucleotides to form 4SU-labelled RNA viral particles. These particles are isolated and used to infect unlabeled cells. Subsequently, 365 nm UV light is used to specifically UV cross-link the viral 4SU-labelled RNPs. The use of 365 nm UV light ensures that normal unlabeled RNPs will not cross-link. After specifically cross-linking the viral RNPs, they are sequence independently purified using a solid phase purification under denaturing conditions and are identified by mass spectrometry. The unique and innovative property of this technique is that the interactome of pre-replicated viruses can be studied specifically. The authors conducted a successful pilot experiment on seven different (+/−)ssRNA virus families, indicating the sequence-aspecific character of the technique. For Chikungunya virus (CHIKV), Kim et al. observed that the newly introduced vRNA molecules are predominantly important until 3 h post-infection before replicated genomes take over the viral processes. Importantly, early viral resistance and susceptibility genes can be identified in this manner, as shown by the authors. Although this technique is developed in a rather specific niche to identify the pre-replicated interactome of RNA viruses, it could be adapted to study the interactome of specific endogenous RNA molecules. In vitro, any type of RNA molecule can be transcribed and 4SU-labelled. These synthetic 4SU-labelled RNA molecules could be delivered to cell cultures by electroporation [71] or packaging in extraneous vesicles [72] to enter the cells by endocytosis. This is dependent upon the ability to transfer enough exogenous 4SU-labelled RNA molecules into the cells. While this approach could be used to study the interactome of theoretically every type of RNA molecule, this still needs to be established in practice.

#### 3.1.3. Phase Separation

Phase separation is a popular principle to separate or concentrate a certain molecular fraction based on its biochemical properties. Although the following techniques (XRNAX, PTex, OOPS) all make use of the same principle, variations introduce subtle differences which can be used to achieve different goals. The general principle depends on the difference in the tendencies of RNA, DNA and proteins to solubilize during the well-established acidic guanidinium thiocyanate-phenol-chloroform extractions protocol (AGPC). Interestingly, although a well-known protocol for decades, it was only recently realized that RNA–protein cross-linked fractions also migrate to one of the specific phases (most often the interphase, except for PTex). This property not only pre-purifies/isolates but also concentrates the RNP fraction, making downstream processes more achievable [21]. Importantly, since these protocols rely on the physicochemical properties of RNPs in general and not on specific RNA sequences or motifs, the whole RBPome will be represented and not a subset, as is the case with RIC.

##### Protein-Cross-Linked RNA eXtraction (XRNAX)

Trendel et al. (2019) recently developed protein-cross-linked RNA eXtraction (XRNAX), as a general first step for the biochemical purification of a crude UV cross-linked RNP fraction. In short, after an AGPC separation, protein–RNA complexes precipitate on the interphase also containing DNA and glycoproteins. After the washing and solubilization of the interphase, the DNA is digested and the RNPs are concentrated by isopropanol precipitation. Non-cross-linked proteins and RNA molecules trapped in the AGPC interphase were also detected in this crude fraction. To solve this issue, a denaturing silica-based cleanup procedure, comparable to the 2C method, downstream of XRNAX was applied to further purify the RNP fraction [21].

##### Orthogonal Organic Phase Separation (OOPS)

Similar to XRNAX, cells containing UV cross-linked RNPs are lysed using AGPC and biphasically separated. However, during the orthogonal organic phase separation (OOPS) protocol, the interphase containing the RNPs is subsequently washed 3 to 4 times by the repeated AGPC separation and precipitation of the interphase. This step releases unbound proteins and RNA molecules caught in the mixture of DNA and RNPs precipitated at the interphase. This facilitates the migration of the molecules to the appropriate phases and minimizes contaminants. Glycosylated proteins share physicochemical properties with RNPs because of their glycosylation and will therefore also co-precipitate on the interphase. Subsequently, the interphase is RNase treated and, in a final AGPC separation step, the released proteins migrate to the organic phase (while glycoproteins and other prepurified contaminants stay at the interphase) and are recovered by methanol precipitation, generating a pure version (96%) of the RBPome [22].

##### Phenol-Toluol Extraction (PTex)

In contrast to OOPS and XRNAX, the Phenol-Toluol extraction (PTex) method [61] makes use of an interplay of two distinct biphasic extractions in tandem, exploiting their physicochemical differences (more specifically described in [23]) to uniquely target RNPs. In the first step, a Phenol-Toluol extraction is performed. Interestingly, this mixture, under neutral pH conditions, directs RNA, proteins and RNPs to the aqueous phase, whereas DNA and lipids are concentrated at the interphase. In the second step, the aqueous phase is subsequently separated under chaotropic and acidic (pH: 4.8) conditions using phenol. During this step, RNA migrates to the aqueous phase, proteins to the organic phase and RNPs to the interphase, then finally precipitated using ethanol. This three-hour protocol highly enriches cross-linked RNPs, containing RNAs as small as 30 bp, while efficiently depleting non-cross-linked proteins and nucleic acids [61]. RNPs containing shorter RNA species such as miRNA could not efficiently be purified, and the author estimates a recovery of 25–30% of the initially cross-linked RNPs, making PTex less applicable if the starting material is scarce.

While XRNAX, OOPS and PTex are based on the same key principle that RNPs precipitate at the interphase of an AGPC separation, they each have their tricks to further efficiently purify this crude interphase fraction into a sufficiently clean and identifiable RBPome fraction. XRNAX uses a downstream denaturing silica-based cleanup step which washes away unbound protein and glycoprotein contaminants, increasing the RNP content from 69% to 89% [21]. OOPS makes use of four consecutive separations of the interphase to increase the percentage of depletion of free protein contaminants before treating the precipitated RNPs with RNase to release the bound proteins. The released proteins migrate to the organic phase in an additional separation step and are now a highly pure (96%) representation of the RBPome [22]. PTex, on the other hand, makes use of a biphasic phenol-toluol prepurification step, removing DNA and lipids before precipitating the RNPs at the interphase of an AGPC step. The lack of DNA during this precipitation step results in a mixture in which free protein contaminants are less likely to be co-precipitating, avoiding these contaminants. An exchange of the key steps in these methods can potentially even further increase the purity of the isolated RNP fractions, and should be further explored experimentally.

### 3.2. Targeting a Specific RNA of Interest to Study Its Interacting Proteins

Unlike isolating an entire group of RNPs as described above, techniques are also available to target a specific RNP of interest. These methods can be categorized into two main groups: approaches using modified/tagged RNA molecules as bait and methods targeting a specific RNA by the use of antisense probes. The modified/tagged based methods fall out of the scope of this review, and this is therefore briefly discussed and not present in the summary Table 2. More information in this regard can be found in the following review [73].

#### 3.2.1. Modified/Tagged RNA Based Methods

Several techniques such as ribotrap [80], tandem RNA affinity purification (TRAP) [81], MS2-biotrap [82] and RNA–protein interaction detection (RaPID) [83] make use of the same principle of introducing a tag in the sequence of the RNA of interest, enabling RNP isolation by affinity purification. Possible tags could be a streptavidin-binding RNA aptamer (4 × S1m) [84], MS2 RNA hairpin [85], a 16-nt hairpin sequence recognized by csy4 [86], etc. The advantages of these techniques are, for example, the potential combination with localization experiments [83] or fewer limitations because of transcript abundance. Although successful, there are some limitations inherent to these strategies. One of the main disadvantages includes the requirement to genetically engineer the RNA. This may alter the secondary structure or functionality and, as a consequence, potentially the composition and formation of RNPs. Additionally, this genetic engineering requirement is limiting the applicability of these methods to a wide range of organisms. In addition, the overexpression of the modified genes may imbalance the cells’ state or alter the interacting RBPs. Overall, the possibility of introducing artefacts due to the non-physiological conditions inherent to these methods is more likely than if using antisense probes. It should be noted that some antisense probe-based methods, such as the use of PPR proteins [78], also require genetically modifying the organism. However, the recombinant protein expressed is not a part of the native RNP complex under investigation, as is the case for the modified/tagged RNA. Introducing non-specific interactions is therefore less likely to occur.

#### 3.2.2. Antisense Probe-Based Methods

##### Peptide Nucleic Acid (PNA)-Assisted Identification of RBPs (PAIR)

**Figure 2:**
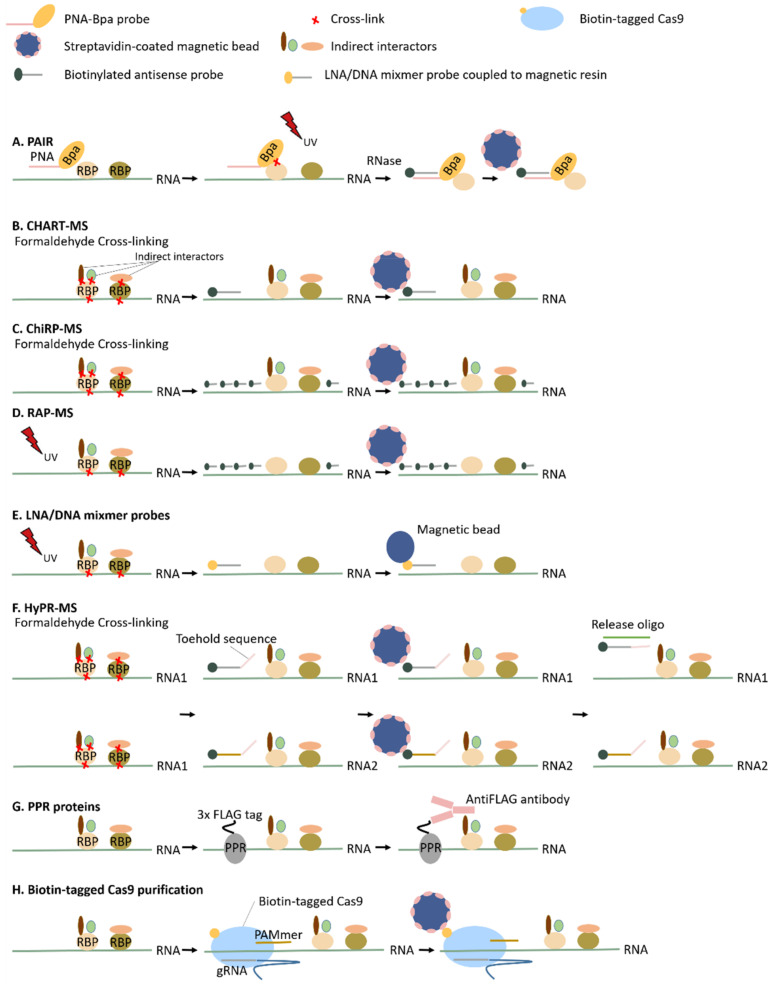
Visual representation of the discussed antisense probe-based methods. Details of following techniques can be find in Section 3.2: (**A**) Peptide nucleic acid (PNA)-assisted identification of RBPs (PAIR), (**B**) Capture hybridization analysis of RNA targets (CHART), (**C**) Chromatin isolation by RNA purification (ChIRP), (**D**) RNA antisense purification (RAP), (**E**) Specific RNP capture with antisense LNA (locked nucleic acid)/DNA mixmers, (**F**) Hybridization purification of RNA-protein complexes (HyPR-MS), (**G**) pentatricopeptide repeat proteins (PPR), (**H**) Biotin-tagged Cas9 purification.

In 2005, Zielinski et al. developed a technique, referred to as the peptide nucleic acid (PNA)-assisted identification of RBPs (PAIR, Figure 2A) [74]. This method was one of the first RNA-centric methods to target RNPs in vivo and was initially used to identify RBPs specifically interacting with the ankylosis mRNA–a panneuronal dendritically localized RNA. The PNA oligomer, a nucleic acid analog with a polyamide scaffold as a substitution for the sugar-phosphate backbone [87], is delivered into the cells by a disulfide-bond-linked cell-penetrating peptide (CPP). Subsequently, the PNA oligomer hybridizes to its complementary RNA target sequence. P-benzoylphenylalanine (Bpa), a photoreactive amino acid adduct, is attached to the hybridized PNA oligomer and, upon UV radiation, Bpa cross-links the nearest RBPs. This PNA-Bpa-RBP complex is isolated using antisense biotinylated oligonucleotides targeting the PNA sequence and streptavidin-coated magnetic beads. As a consequence of the maximum distance between Bpa and the RBP (<4.5 Å), this technique enables not only to specifically target the RBPs of an RNA of interest but also identifies RBPs interacting with a specific region of the RNA. However, to identify all RBPs interacting with a single RNA molecule, it is necessary to include several PNA oligomers distributed along the RNA (+/−every 25 base pairs), increasing the costs of the experiment significantly. Due to secondary structures of the RNA, not all regions will be accessible for the PNA oligomer and, as a result, this technique is not advisable when identifying the whole interactome of a specific RNA.

##### Capture Hybridization Analysis of RNA Targets (CHART), Chromatin Isolation by RNA Purification (ChIRP) and RNA Antisense Purification (RAP)

Inspired by chromatin immunoprecipitation (ChIP), where protein-DNA interactions are investigated, Simon et al. developed in 2011 the Capture Hybridization Analysis of RNA Targets (CHART) [87]. Simultaneously with the development of CHART, Chu et al. developed chromatin isolation by RNA purification (ChiRP-seq) [88]. Both methods were initially developed to enrich lncRNAs together with their interacting DNA for the investigation of genomic binding sites, which are thought to be regulators of the chromatin state due to their known interaction with chromatin modification complexes [89,90]. Although they initially focused on RNA–DNA interactions, these methodologies are easily extended to the study of RNA-associated proteins by coupling the purification of the specific RNA targets to mass spectrometry analysis. This was subsequently shown by West et al. (2015), who applied CHART-MS to identify the RBPs of the lncRNAs NEAT1 and MALATI1 (Figure 2B) [75], and Chu et al. (2015) who applied ChiRP-MS to identify the specific interactors of Xist lncRNA (Figure 2C) [10]. Additionally, the RNA antisense purification method (RAP) developed in 2013 by Engreitz et al. to investigate the RNA occupancy of the Xist lncRNA during the inactivation of the X chromosome [91] was modified in 2015 by the same group to isolate lncRNAs and their associated proteins, referred to as RAP-MS (Figure 2D) [11]. This technique was first used to elucidate specific interactors of the Xist lncRNA [11]. Although conceptually similar, because of the cross-linking of lncRNAs to their interaction partners and the subsequent purification by the use of biotinylated complementary oligomers and streptavidin-coated magnetic beads, there are clear differences between the above techniques. One technical difference involves the number of probes used. For CHART, a mixture of only three probes is used, whereas for both ChiRP and RAP the probes are in such quantity that the entire length of the RNA is tiled. Consequently, for CHART, the accessibility for the probes (probes cannot bind dsRNA or RNA protected by a protein) has to be determined beforehand based on RNase H assays, whereas for ChiRP and RAP there is no real necessity to explore the RNA structure and accessibility in advance. The use of a large number of probes, however, can result in higher background contamination. Not only the number of probes but also the length of the probes differs between the different approaches. Both CHART and ChiRP make use of short probes (around 20-nt), whereas RAP uses probes of around 120 nt, possibly resulting in a higher specificity. Both RAP and ChiRP were used to identify the interaction partners of lncRNA Xist. For RAP, a yield of 70% of the endogenous RNA was retrieved, whilst 60% was retrieved for ChiRP. Although similar yields were achieved, there is a remarkable difference in the number of identified interacting proteins. ChiRP identified 81 proteins interacting with Xist lncRNA, whereas RAP only identified 10. RAP uses UV cross-linking, known to only induce RNA–protein interactions, whereas ChiRP (and CHART) uses formaldehyde cross-linking, inducing protein–protein cross-links also. This may be a plausible explanation for the observed difference. As mentioned, common to all three approaches is the use of biotinylated oligomers. In order to avoid the co-purification of rare naturally biotinylated proteins by the streptavidin-based purification of the probes, lysates are pre-cleared for biotin-linked molecules by incubating and removing streptavidin-coated magnetic beads before hybridizing the antisense biotinylated probes. The pre-clearance step, of course, increases the costs of the experiment [10,11,75].

##### Specific RNP Capture with Antisense LNA (Locked Nucleic Acid)/DNA Mixmers

Unlike CHIRP-MS and RAP-MS [10,75], the specific RNP capture method developed by Rogell et al. in 2017 is not based on the so-called “tiling approach”. Tiling approaches may be advisable when targeting RNA with impaired integrity, whereas they are not the optimal strategy to investigate specific regions or different transcript isoforms. Instead of using a quantity of probes to cover the entire stretch of the RNA of interest, Rogell et al. made use of a single LNA/DNA mixmer probe (Figure 2E) [76]. This method was inspired by RIC designed to capture the mRNA interactome by the use of UV cross-linking and antisense oligo(dT) beads [3,4]. To adapt this protocol to the target, only the RBPs of a specific RNA of interest, 20-mer antisense LNA/DNA mixmer probes, were used instead [76]. LNA nucleotides are DNA analogs with an extra 2′-*O*-CH2-4′ bridge, increasing the probes’ melting temperature up to 10 °C per LNA nucleotide in the mixmer sequence [92]. The LNA/DNA mixmer probes are covalently linked to a magnetic resin resulting in the isolation of the RNA of interest together with its interaction partners. The main advantage of the increased melting temperature is the resulting increased hybridization specificity. As a consequence, the use of only one probe complementary to the target RNA is considered to be sufficient. However, possibly because of this single probe approach, the recovery is only around 20%, rendering this technique less optimal for the isolation of low-copy-number transcripts, such as lncRNAs. The efficacy of this technique was proven in both in vitro (interaction *Drosophila* Sex lethal (Sxl) and the male specific lethal-2 (msl-2) mRNA) and in vivo (ribosomal 18S and 28S rRNAs) systems [76]. For the in vivo rRNP isolation, only a part of the expected ribosomal proteins was identified. This is potentially due to the inefficient UV cross-linking of dsRNA–protein interactions [76]. As described in Section 2.3, the use of methylene blue could resolve this problem. Additionally, the complementary use of formaldehyde cross-linking can increase the identification of these dsRNA binders.

##### Hybridization Purification of RNA–Protein Complexes Followed by Mass Spectrometry (HyPR-MS)

In 2018, Spiniello et al. reconsidered the existing framework to capture specific RNPs, as described for CHART-MS, CHIRP-MS, RAP-MS and LNA/DNA mixmer probes [10,11,75,76]. They established the hybridization purification of RNA–protein complexes (HyPR-MS), a cost-effective protocol to target multiple lncRNAs in a single experiment (Figure 2F) [77]. Two to three antisense, biotinylated probes per target are designed with Mfold software [93] to hybridize with MALAT1, NEAT1 and NORAD lncRNAs subsequent to formaldehyde cross-linking. These probe–lncRNA-RBP complexes are isolated with streptavidin-coated magnetic beads [77]. After the simultaneous capture of multiple targets, each target is released in a sequence-specific manner, referred to as the toehold-mediated release [94,95]. Each probe harbors an additional 8-nt sequence, which does not hybridize the target, called the toehold sequence. Whenever a release oligonucleotide (RO), completely complementary to the probe (toehold sequence + target sequence) is added, a probe-RO hybrid is formed. This complex is thermodynamically more favorable than the probe–lncRNA hybrid, resulting in the solubilization of the target RNA. By capturing the different targets from the same cell lysate, the sample and background variability is almost reduced to zero. Advantageously, labor and costs may be reduced by conducting only one multiplexed experiment [77].

##### Expression of Proteins That Are Guided to or Recognize Specific RNA Molecules (PPR Proteins and Biotin-Tagged Cas9 Purification)

A recently established method by McDermott et al. (2019) differs by using artificial pentatricopeptide repeat proteins (PPR) rather than antisense oligonucleotide probes to study in vivo RNPs (Figure 2G) [78]. PPR proteins, one of the largest protein families in plants, are known to bind RNA in a sequence-specific manner. This specific interaction is based on the presence and position of two amino acids in all helical tandem repeats [96,97,98,99,100,101,102,103]. By altering these amino acids, any RNA of interest can be targeted [100,103,104,105]. McDermott et al. were the first to develop such an artificial PPR protein-based RNP capture in vivo in *Arabidopsis thaliana* to specifically target chloroplast psbA mRNA. The PPR proteinsused contain an N-terminal chloroplast targeting sequence and a C-terminal 3xFLAG tag. The AntiFLAG antibody was used to co-immunoprecipitate the PPR protein-psbA complex (harboring interacting RBPs). The efficacy of this model for cytoplasmic RNAs is yet unknown considering PPR proteins are most often found in chloroplasts and mitochondria in nature [78]. Another method worth mentioning which uses protein-mediated RNP purification is the RNA-guided, biotin-tagged Cas9 purification of specific RNPs (Figure 2H) [79]. In a native context, Cas9 will only recognize and interact with dsDNA. O’Connell et al. exploited the well-known Cas9 RNA-guided DNA endonuclease system and established a Cas9 RNA-guided ssRNA-binding system. Through the use of an extended PAMmer DNA oligonucleotide, Cas9 is able to bind with high affinity to any ssRNA of interest complementary to its guide RNA (gRNA). The biotinylation of Cas9 enables purification with streptavidin-coated magnetic beads. For both PPR protein-based RNP capture and biotin-tagged Cas9 purification, an important consideration when using proteins to target a specific RNA is that the system relies on the integrity and functionality of the protein, so one cannot use denaturing purification conditions.

## 4. Overcoming Cost, Yield and Specificity Challenges by Combining Methods

Whereas UV cross-linking is a very specific cross-linking technique, it lacks efficiency, which is its main drawback. It is estimated that only 1–5% of the RNPs are actually cross-linked, leaving 95–99% free. Nevertheless, we believe that because of its specificity and ease of use, UV cross-linking is still the most suitable and general strategy to use in RNA-centric approaches. Therefore, we will only elaborate on methodologies that make use of UV cross-linking. This does not mean that our rationale cannot be applied to chemical cross-linking or native experiments.

Peptides, unlike DNA/RNA, cannot be amplified. Until a technique is found to do so, this means that what you isolate is what you have to detect. Depending on the goal, overcoming the detection limit of mass spectrometry can be challenging. Rogell et al., making use of LNA/DNA mixmer probes, estimated that the amount of a captured single RNA target should be around 2 µg to reach the mass spectrometry detection limit [106]. These numbers were calculated for the capture of highly abundant ribosomal RNPs. When low-copy-number RNA is targeted, extensive amounts of starting material and (often expensive) chemicals have to be used to reach RNP concentrations detectable with mass spectrometry. Different strategies to overcome the detection limit, besides scaling-up, can be based on reducing the background to make detection easier. Additionally, making the capture technique more efficient and thus generating more detectable targets per cell is a possibility. We believe that making use of a combination of previously described methodologies can increase the capture performance on all these levels.

**Figure 3:**
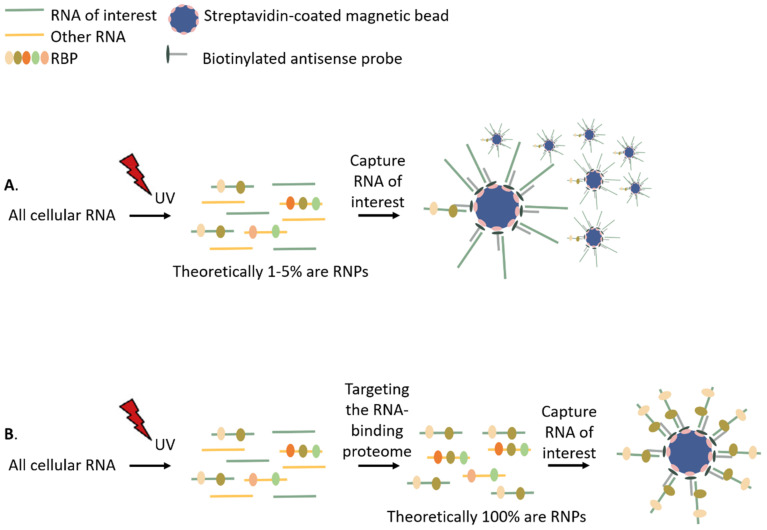
Non-cross-linked (free) RNA interferes with efficient RNP capture. (**A**) Most of the RNA-targeting techniques capture RNA from a cross-linked lysate. Due to the low UV cross-linking efficiency, only 1–5% of the captured RNA molecules will be linked with an RBP. (**B**) If, after UV cross-linking, the RNPs are separated from free RNA molecules, theoretically all the captured RNA is linked with an RBP. As a result, a smaller number of probes/beads is necessary to isolate the same amount of RBPs. In this figure, antisense, biotinylated probes and streptavidin-coated magnetic beads are depicted. However, similar mechanisms are true when using oligo (dT) probes, azide biotin, PNA-Bpa probes, LNA/DNA mixmer probes with a coupled magnetic resin, biotin-tagged Cas9 or PPR proteins with a C-terminal 3xFLAG tag.

At first sight, non-cross-linked (free) RNA is not perceived as a problematic contaminant in RNP isolation studies. Indeed, in theory, it harbors no protein contaminants and thus will not affect the mass spectrometry analysis. However, we believe that removing free RNA is one of the major protocol improvements to make. Free RNA not only contaminates the isolate by dragging in transiently bound unlinked proteins but also competes with RNPs to be isolated. Knowing that most of the RNA is not cross-linked, this generates strong competition and makes methods such as (e)RIC, RAP-MS, specific capture with antisense LNA/DNA mixmer probes, RICK and CARIC less efficient (Figure 3A). These methodologies isolate their targets from lysate using streptavidin- or antisense coated beads without discrimination between RNPs and free RNA. Because most of the present RNA is non-cross-linked and, in addition, lacks steric hindrance for probes in comparison to RNA cross-linked to proteins, an excess amount of non-cross-linked RNA in the isolate is expected. This will lower the capture efficiency of RNPs per bead drastically.

As described, organic phase separation methods such as XRNAX and PTex isolate RNPs at the interphase. Importantly, besides isolating the whole RBPome, these techniques also separate the RNPs from free RNA and proteins, leaving a purer RNP mixture, and are comparable with the strategy of the Tandem RNA Isolation Procedure (TRIP) [106]. Using this organic phase separation fraction instead of the classical lysate, one can concentrate the cross-linked RNP fraction from ±5% to theoretically 100% dissolved in a buffer of choice. This dramatically increases the capture/isolation efficiency (Figure 3B) and reduces costs and labor. Besides being free from non-cross-linked RNA and proteins, the organic phase separation fraction also lacks the majority of cell debris present in input lysates such as lipids, proteinase/RNase, radicals, biotin-containing molecules, salt, etc. If these contaminants are still present, they could interfere with the efficiency and integrity of the isolation procedure. Depleting these contaminants expands the number of buffers and conditions that can be used to optimize capture protocols (RAP-MS, (e)RIC, specific capture with antisense LNA/DNA mixmer probes), but also chemical reactions (RICK, CARIC). This could result in a higher yield, lower background and better integrity of the target RNPs. As an example, RICK/CARIC and RAP-MS in combination with organic phase separation will be discussed.

McHugh et al., 2015 successfully used RAP-MS to identify interacting proteins of the lncRNA Xist. Although already proven to work on high-copy-number RNPs, such as ribosomal and spliceosomal complexes, they were the first to successfully use this technique on a low-copy-number RNA molecule, highlighting its potential. After such success, one would expect an explosion of similar studies. To the best of our knowledge, only a few papers appeared that specifically identify interacting proteins to a single specific RNA target [14,107,108,109].

This suggests the lack of a scalable, cost-efficient and easy to execute protocol. To give an idea of the used scale and cost, 8 × 10^10^ cells per sample (8 samples in total) and 76.8 mL of MyOne streptavidin C1-coated dynabeads representing a value of approximately 24,000 euros (at the time of writing) were used. Half of the amount of dynabeads was used to pre-clear the lysate from native biotin-linked molecules. This scale and cost exceed the costs of an appealing, routinely usable procedure for wide adoption. As discussed above, an organic phase separation pre-step already eliminates most of the free RNA, which will theoretically increase the RAP-MS methods’ yield by ~20 times (from 5% to theoretically 100% target RNA harboring proteins). In addition, PTex needs 100 times less starting material in comparison with RIC to achieve a similar goal. Along with the higher RNP capture efficiency, biotin-linked molecules are also removed. This makes a biotin pre-clearing step unnecessary, reducing the experimental time and amounts of expensive materials. If the above assumptions are correct, a similar RAP-MS experiment based on an organic phase separation fraction would be possible for the assumed cost of 600 euro in streptavidin beads, which makes it much more appealing to be routinely used. Additionally, other techniques which do not discriminate between free RNA and their RNP target can benefit from this organic phase separation pre-purification step such as (e)RIC, PPR protein, HyPR-MS, CHART-MS and ChiRP-MS, but this still needs to be established in practice.

RICK and CARIC enable the highly interesting feature of making a distinction between RNPs comprising newly transcribed RNA relative to older complexes. This characteristic could enable the dynamic mapping of RNPs upon different biological/chemical conditions. A challenging part of the click-based approach is to perform an efficient click reaction to link biotin to the 5-Ethynyl-uridin (EU) analogs without causing RNA degradation due to formed Cu(I) ligands and lysate debris. Although Huang et al. (2018) already use ultrafiltration tubes to pre-purify the RNP fraction, we think that using an organic phase pre-separation could minimize RNP degradation during the click reaction. This is due to the increased probability of optimal reaction conditions and the lack of RNase/proteinase and other interfering lysate components. Along with the possibility to control the reaction conditions, naturally present biotin-linked proteins and metabolites are removed. Therefore, the lysate will not have to be pre-cleared before performing the streptavidin-coupled bead capture, reducing experimental time and expensive chemicals.

Together, the above-discussed feature of removing free RNA results in a more efficient capture experiment on the same amount of input material. When scaling up an experiment, the required volumes could exceed practical sizes. The organic phase pre-separation can easily handle big volumes to process down to a much smaller RNP fraction. This efficiently tackles potential scaling problems on the condition that starting material is not limited. We believe that this property, together with separating RNPs from free RNA and free proteins, will make organic phase pre-purification a gold standard in all facets of future RNP research.

**Figure 4:**
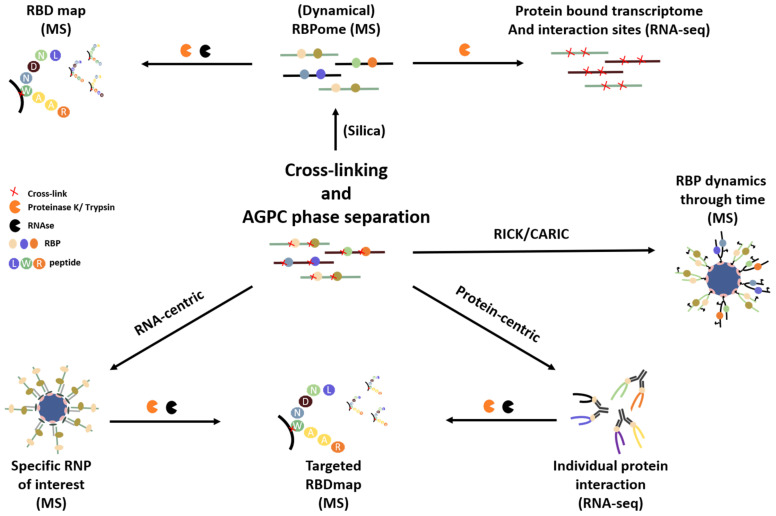
AGPC phase separation as a starting point for downstream processes: By using an AGPC phase separation pre-purification step, challenging protocols in terms of yield, scale, purity and quality can be simplified. Both the RBPome as its dynamics upon a biological stimulus can be studied by quantitative mass spectrometry directly from this fraction or after an additional purification step using silica columns. Starting from the RBPome fraction the RBD map (which amino-acid of the protein interacts with the RNA molecule?), the protein bound transcriptome (how does the transcriptome change its association with proteins upon a stimulus?) and the exact interaction site at nucleotide resolution can be explored. The AGPC fraction will also serve as a good starting point to enhance more specific protein-centric (CLIP) and RNA-centric (RIC, RAP, CARICK and RICK) approaches to identify proteins/RNA that interact with one specific or a subset of proteins/RNA molecules. Figure inspired by [10,11].

While this review is focused on RNA-centric methods, starting from a clean RNP fraction can also be very helpful to enhance downstream protocols to elucidate ribonome transcriptomics, RBP mapping [42,43,110], RBP footprinting [111] and protein-centric approaches such as CLIP-seq [40], highlighting its broad applicability (Figure 4).

## 5. Standardized Data Analysis and Visualization

The study of the ribonome is a rapidly evolving field. With the new organic phase separation methods, the field is pushed even further. Multiple methodologies are being developed in parallel which make use of different experimental conditions and choices. Experimental differences occur at all stages, starting with the choice of cell or tissue type, cross-linking methods and doses, use of different control sampling, mass spectrometry equipment and bioinformatic proteomic approaches. Lacking a clear consensus on the experimental set-up and on how to analyze and visualize data makes comparisons between methods difficult.

Both ChiRP-MS and RAP-MS were independently performed to study the interacting proteins of Xist lncRNA. Due to the similar research question, these techniques are a good example to illustrate that differences in experimental set-up may greatly influence outputs. For ChiRP-MS, 81 interacting proteins were identified, whereas only 10 were identified for RAP-MS. The identified proteins for both techniques show an overlap of eight. ChiRP-MS makes use of human HeLa cell lines, whereas RAP-MS uses a mouse pSM33 ES cell line. Differences in cell type may contribute to the different identified RBPs [10,11]. In addition, even when the same cell type is used, differences in growth conditions such as glucose deprivation potentially influence the result [54]. Furthermore, the cross-linking method may play a role in the experimental output. Formaldehyde cross-linking, used by ChiRP-MS, also results in protein-protein cross-links, whereas UV cross-linking, used by RAP-MS, is known to be more specific. This potentially explains the numerical difference in proteins identified [10,11]. There are even examples where different UV wavelengths (254 nm and 365 nm) result in a rather complementary output instead of a fully overlapping one [24].

Another example is the comparison of three mRNA interactome capture experiments conducted in Arabidopsis, which only share an overlapping set of 79 proteins of the total 1815 proteins detected. As stated by Köster et al., apart from the differential mass spectrometry data analysis, the diverse developmental stages of the plants examined in each of these studies may contribute to the different outputs [59]. Other differences may be caused by the different controls used to eliminate background contamination. These can be, for example, a non-targeting scrambled probe, the capture of a reference RNA, an RNase-treated or non-cross-linked sample which all may result in different quantitative proteomics outputs. Generally, a non-cross-linked control sample is considered as the preferred control for the elimination of background contamination. This is due to the properties specific for the target RNA to potentially bind certain proteins non-specifically. However, a non-cross-linked control can only be included when the purification is established in denaturing conditions to avoid false negatives from being introduced. If a non-cross-linked control is not optional, a combination of controls should be included to increase the reliability.

Furthermore, when analyzing mass spectrometry data, different false discovery rates, peptides required for protein identification, number of biological replicates, protein enrichment fold cut-offs, mass spectrometry equipment, quantitative proteomics approaches, etc., also contribute to a more difficult comparison of the outputs of each experiment and method.

A final important aspect is the evaluation of the efficacy of the experiment. This is often presented as the relative abundance of the target RNA compared to a reference RNA after the experiment in contrast to its ratio in the input sample, meaning that the levels of the target RNA after purification are normalized to the levels in the input sample. This is known as the fold-enrichment of the target RNA. By comparing to the input sample, the target RNA may be enriched several-fold; however, it is possible that in absolute numbers, the target RNA, when capturing a low-copy-number RNA for example, may not exceed the abundance of the reference and other contaminating RNA molecules. In quantitative proteomics approaches, contamination will be indeed identified as being contamination. However, it is possible, especially for low-copy-number RNA, that protein contamination, if too abundant, complicates the detection of the low-abundant target proteins due to the occupation of the ion trap sampling space by these contaminants. Additionally, the often-used semiquantitative approaches based on spectral counting have troubles in reliably identifying low-abundance proteins due to inherent variations [112]. As a result, in our opinion, RNA fold-enrichment should always be combined with RNA-seq or bioanalyzer analysis to reliably address sample purity in order to ensure competition between the target protein and the contaminants being detected is avoided as much as possible. However, such analyses again contribute to the cost of the experiment. As an alternative to evaluate the efficacy, we propose to calculate the ratio of the number of molecules of the reference RNA per number of molecules of the target RNA, representing the relative abundance of contaminating RNPs, only after the capture step.

## 6. Conclusions

The complex life of RNPs is still far from being a fully understood system. In the last decade, a wealth of methods has been developed to address pieces of this puzzle. Each method, however, has its own characteristics and strengths, but also there are clear technical limitations holding back the possibility of studying RNPs with a clear and general approach. We believe that the arrival of the phase separation methods, such as XRNAX, OOPS and PTex, will catalyze the former techniques into more effective tools to study RNPs. Due to both experimental and bioinformatics differences in set-up, it is often difficult to compare and evaluate techniques, often resulting in different outputs for similar research questions. Therefore, these methods can be considered often rather complementary than equal, rendering it more important to choose the technique tailored to the desired output.

## Figures and Tables

**Table 1:** Overview of techniques to isolate the RNA-bound proteome to study RNP dynamics.

Method	RNA Target	Advantage	Disadvantage	Cell Type	Cell Number
Affinity-Based Separation					
RIC [3,61]	Poly(A) tailed RNA	Isolates only mRNA complexes (if subset is of interest) Widely used protocol	Isolates only mRNA complexes Low signal-to-noise ratios Additionally, co-purification of non-cross-linked (free) RNA Can purify off-target RNA containing poly(A) stretches within its sequence	All poly(A) tailed containing organisms	7500 cm^2^ HeLa cells [3] 10^7^ cells [39] 10^8^ cells [63]
e/cRIC [7,9]	Poly(A) tailed RNA	Isolates only mRNA complexes (if subset is of interest) Better signal-to-noise ratios than RIC	Isolates only mRNA complexes Additionally, co-purification of free RNA Can purify off-target RNA containing poly(A) stretches within its sequence	All poly(A) tailed containing organisms	1–1.3 × 10^8^ cells [7] 3 × 15 cm dishes at 80% confluence [9]
CARIC [64]	Newly transcribed RNA	All RNA types RNP monitoring through time	Use of nucleoside analogs Potential co-purification of naturally biotinylated proteins Additionally, co-purification of free RNA	Limited to cell cultures receptive for nucleoside analogs	4 × 10^7^ cells
RICK [65]	Newly transcribed RNA	All RNA types RNP monitoring through time	Use of nucleoside analogs Potential co-purification of naturally biotinylated proteins Additionally, co-purification of free RNA	Limited to cell cultures receptive for nucleoside analogs	Not specified
Solid Phase Separation					
2C [66]	All RNPs	Fast and cost-effective method	Contamination of both free protein and free RNA Dependent on the scale of the silica columns A nucleotide size limitation can occur inherent to silica matrices	All cell types and tissue	Not specified
(PAR)-TRAPP [43]	All RNPs	Cost-effective method Scalable protocol	DNA is co-eluted Additionally, co-purification of free RNA A nucleotide size limitation can occur inherent to silica matrices	All cell types and tissue	750 mL of media containing cells at an OD600 of 0.5
VIR-CLASP [67]	Pre-replicated viral RNPs	Study of early-stage viral infection Theoretically adaptable to every type of in vitro transcribed RNA molecule Cost-effective method	The current field of application is a highly interesting but small niche SPRI beads can have size-selective artefacts	Limited to cell cultures receptive for nucleoside analogs	15 cm^2^ of cells
Organic Phase Separation					
XRNAX [21]	All RNPs	All RNA types Little free RNA Cost-effective method Easily scalable Good starting point for more specific techniques	Glycoproteins and RNA–protein adducts cannot be distinguished Technically challenging Crude fraction	All cell types and tissue	1 × 10^8^ cells
OOPS [22]	All RNPs	All RNA types Cost-effective method Easily scalable	Technically challenging Cannot be used as a starting point for more specific techniques	All cell types and tissue	28.2 cm^2^ of 90% confluence
PTex [23]	All RNP >30 bp	All RNA types Little free RNA Cost-effective method Easily scalable Good starting point for more specific techniques	Glycoproteins and RNA–protein adducts cannot be distinguished Technically challenging 25–30% recovery	All cell types and tissue	2 × 10^6^ cells

**Table 2:** Overview of techniques to target a specific RNA of interest to study its interacting proteins.

Method	Advantage	Disadvantage	Cell Number
Antisense Probe-Based Methods			
PAIR [74]	Direct interactors RBPs of a specific region Different transcript isoforms Denaturing purification conditions	Difficult to study whole interactome of a specific RNA Potential co-purification of naturally biotinylated proteins Costs of CPP-PNA-Bpa probes	2 × 10^6^–1 × 10^7^ cells
CHART-MS [75]	Different transcript isoforms Denaturing purification conditions	Direct and indirect interactors RNase H assay to determine accessibility of the probes Potential co-purification of naturally biotinylated proteins	8 × 10^7^ cells
ChiRP-MS [10]	No prior knowledge of the RNA structure and accessibility required Capture of RNA with impaired integrity Denaturing purification conditions	Direct and indirect interactors Large number of probes may result in higher background contamination Potential co-purification of naturally biotinylated proteins Costs of large number of probes	1–5 × 10^8^ cells depending on the cell type
RAP-MS [11]	Direct interactors No prior knowledge of the RNA structure and accessibility required Long probes may result in high specificity Capture of RNA with impaired integrity Denaturing purification conditions	Large number of probes may result in higher background contamination Potential co-purification of naturally biotinylated proteins Costs of large number of probes Cost of synthesizing long probes	2 × 10^7^ cells
LNA/DNA mixmers [76]	Direct interactors Different transcript isoforms Increased hybridization specificity Denaturing purification conditions	Cost of LNA-containing probes Use of only one probe results in RNA integrity to be critical 20% recovery Less optimal for low-copy-number transcripts	2000 cm^2^ of HeLa cells at 70% confluence
HyPR-MS [77]	Cost- and labor-effective protocol Reduced sample and background variance Different transcript isoforms Denaturing purification conditions	Direct and indirect interactors Potential co-purification of naturally biotinylated proteins	1 × 10^8^ cells
PPR proteins [78]	Different transcript isoforms	Direct and indirect interactors Efficacy only proven in chloroplasts and mitochondria Restriction to use denaturing purification conditions	Chloroplasts isolated from 40 g tissue
Biotin-tagged Cas9 purification [79]	Different transcript isoforms	Direct and indirect interactors Restriction to use denaturing purification conditions Potential co-purification of naturally biotinylated proteins	5x10^6^ cells
